# Cardiotrophin-Like Cytokine Factor 1 Exhibits a Myeloid-Biased Hematopoietic-Stimulating Function

**DOI:** 10.3389/fimmu.2019.02133

**Published:** 2019-09-10

**Authors:** Sarah Pasquin, Aurélie Tormo, Jessica Moreau, Véronique Laplante, Mukut Sharma, Jean-François Gauchat, Moutih Rafei

**Affiliations:** ^1^Département de Pharmacologie et Physiologie, Université de Montréal, Montreal, QC, Canada; ^2^Immuni T, Montreal, QC, Canada; ^3^Renal Division, KCVA Medical Center, Kansas City, MO, United States; ^4^Département de Microbiologie, Infectiologie et Immunologie, Université de Montréal, Montreal, QC, Canada; ^5^Programme de Biologie Moléculaire, Université de Montréal, Montreal, QC, Canada; ^6^Department of Microbiology and Immunology, McGill University, Montreal, QC, Canada

**Keywords:** cardiotrophin-like cytokine factor 1, interleukin-6, LSK cells, myelopoiesis, bone marrow transplantation

## Abstract

Cardiotrophin-like cytokine factor 1 (CLCF1) is secreted as a complex with the cytokine receptor-like factor 1 (CRLF1). Syndromes caused by mutations in the genes encoding CLCF1 or CRLF1 suggest an important role for CLCF1 in the development and regulation of the immune system. In mice, CLCF1 induces B-cell expansion, enhances humoral responses and triggers autoimmunity. Interestingly, inactivation of *CRLF1*, which impedes CLCF1 secretion, leads to a marked reduction in the number of bone marrow (BM) progenitor cells, while mice heterozygous for *CLCF1* display a significant decrease in their circulating leukocytes. We therefore hypothesized that CLCF1 might be implicated in the regulation of hematopoiesis. To test this hypothesis, murine hematopoietic progenitor cells defined as Lin^−^Sca1^+^c-kit^+^ (LSK) were treated *in vitro* with ascending doses of CLCF1. The frequency and counts of LSK cells were significantly increased in the presence of CLCF1, which may be mediated by several CLCF1-induced soluble factors including IL-6, G-CSF, IL-1β, IL-10, and VEGF. CLCF1 administration to non-diseased C57BL/6 mice resulted in a pronounced increase in circulating myeloid cells, which was concomitant with augmented LSK and myeloid cell counts in the BM. Likewise, CLCF1 administration to mice following sub-lethal irradiation or congeneic BM transplantation (BMT) resulted in accelerated LSK recovery along with a sustained increase in BM-derived CD11b^+^ cells. Altogether, our observations establish an important and unforeseen role for CLCF1 in regulating hematopoiesis with a bias toward myeloid cell differentiation.

## Introduction

Hematopoiesis is a tightly regulated process involving numerous factors controlling the balance between hematopoietic stem cells (HSC) self-renewal and lineage commitment (1). Besides the role played by HSC-specific transcription factors, cytokines of the IL-6 family have been shown to exert important functions in the regulation of HSC biology. This is exemplified by the fact that IL-6, which is produced by BM-resident mesenchymal stem cells, acts synergistically with IL-3 to induce HSC and progenitor cell proliferation (2, 3). Consistent with this notion, *Il6* disruption in mice decreases the levels of committed progenitors and colony forming units (4), while inactivating *IL6ST*, the gene coding for the common IL-6 family receptor chain gp130 is embryonically lethal due to constrained hematopoietic development (5). In addition to IL-6, a role for leukemia inhibitor factor (LIF; another member of the IL-6 cytokine family) has been also proposed for stem cell survival and proliferation as its administration to mice increases the counts of megakaryocyte progenitor cells both in the BM and spleen (6, 7).

CLCF1 is another member of the IL-6-type cytokine and is expressed in lymph nodes, spleen and by circulating lymphocytes (8–10). In order to be efficiently secreted, CLCF1 needs to form a composite cytokine with CRLF1 (11). CLCF1 is believed to signal through the heterodimerization of the signaling chains LIFRβ and gp130, which suggests similar overlapping functions with LIF (11). So far, CLCF1 is considered as a neurotrophic factor because of its implication in the survival and development of motor neurons (12). Alike other members of the IL-6 cytokine family however, CLCF1 exerts immunomodulatory functions such as supporting B-cell expansion and humoral responses (9). Of note, CLCF1 overexpression in mice leads to splenomegaly, whereas mice heterozygous for *CLCF1* display lower circulating leukocyte counts (9). Among other severe phenotypes, mice lacking *CRLF1* display a strong decrease in hematopoietic progenitor cell numbers (13). These observations led us to examine whether CLCF1 exerts an effect on hematopoiesis. We report in this study a potent role for CLCF1 on hematopoietic multipotent progenitor cell proliferation *in vitro* with a myeloid-biased hematopoiesis in both healthy animals and following immunoablation.

## Materials and Methods

### Experimental Animals

Female C57BL/6 (H2-K^b^) and B6.SJL (H2-K^b^) mice (6–8 weeks of age) were purchased from the Jackson Laboratory (Bar Harbor, ME) and housed at the animal facility of the Institute for Research in Immunology and Cancer (Montreal, QC). All experimental procedures were conform to the Canadian Council on Animal Care guidelines and approved by the Animal Ethics Committee of Université de Montréal.

### LSK Cell Culture Protocol

Production, purification and quantification of recombinant murine CLCF1 was conducted as previously described (14). BM cells were isolated by flushing femurs and tibias of 8 weeks old C57BL/6 female mice followed by red blood cell lysis using an ammonium-based buffer. BM-derived white blood cells were then resuspended at 5 × 10^5^ cells/ml in RPMI media supplemented with 10% FBS, 10 mM Hepes, 2 mM l-glutamine, 100 U/ml penicillin, 100 μg/ml streptomycin, and 0.1 mM β-mercaptoethanol. Cells were stimulated with recombinant murine CLCF1 or murine IL-6 (at the indicated concentrations) and incubated at 37°C for 24, 48, or 72 h. Stimulated cells were then harvested and stained in PBS containing 0.1% BSA using V450- conjugated -anti-Lineage Cocktail, PercPCy5.5-conjugated anti-CD117 (c-Kit), APC-Cy7 conjugated anti-Ly-6A/E (Sca1) (all purchased from BD Bioscience, Mississauga, ON), PE-conjugated anti-CD48 (ebiosciences, ThermoFisher Scientific Inc., Burlington, ON), Alexa Fluor 647-conjugated anti-CD150 (clone TC15-12F12.2, Biolegend, Cedarlane, Burlington, ON), Super Bright 600-conjugated anti-CD16/CD32 (ThermoFisher Scientific Inc.), FITC-conjugated anti-CD41a (ThermoFisher Scientific Inc.) and PE-Cy7-conjugated anti-CD105 (clone MJ7/18, BioLegend). Fluorescence was quantified with a FacsCanto II flow-cytometer (BD Bioscience).

### Analysis of LSK Cell Functionality Using Colony Forming Unit Assay

BM cells were isolated and cultured as described above in the absence or presence of 100 ng/ml of CLCF1 for 24 h. To assess clonogenic progenitor frequencies, 3 × 10^4^ of freshly isolated BM cells or equivalent volumes of BM cells cultured for 24 h were mixed with methylcellulose-based complete medium (MethoCult™ GF M3434, Stemcell Technologies, Vancouver, BC) and incubated at 37°C for 7 days following the recommendations of the manufacturer. Cultures pictures were obtained using a Zeiss Axio Observer.Z1 microscope (Carl Zeiss Canada Ltd, Toronto, ON).

### Induction and Analysis of LSK Cell Proliferation *in vitro*

BM cells were cultured for 24 h as described above in presence of PBS, 10 or 100 ng/ml of CLCF1. Cells where further incubated with 10 μM of 5-ethynyl-2′-deoxyuridine (EdU) for 18 h following manufacturer instructions (Click-it EdU Alexa Fluor488 flow cytometry assay kit, ThermoFisher Scientific Inc., Burlington, ON). Cells were washed then stained with V450- conjugated -anti-Lineage Cocktail, PercPCy5.5-conjugated anti-CD117 and APC-Cy7 conjugated anti-Ly-6A/E (all purchased from BD Bioscience) prior to fixation and permeabilization. Prepared cells were then incubated with Click-it reaction cocktail for 30 min and the fluorescence quantified by flow-cytometry.

### STAT3 Tyrosine Phosphorylation Assays

Freshly isolated BM cells were stained with anti-Lineage Cocktail, anti-CD117 and anti-Ly-6A/E and resuspended at 1 × 10^8^ cells/ml in sorting buffer (PBS supplemented with 1 mM EDTA, 25 mM Hepes, and 1% FBS). LSKs were sorted using BD FACS Aria high-speed cell sorter (BD Bioscience). Both WBM cells and sorted LSKs were stimulated for 15 min at 37°C with 100 ng/ml of IL-6 or CLCF1. Cells were fixed with 2% formaldehyde for 10 min at room temperature and permeabilized on ice with 90% methanol for 30 min prior to staining with Alexa Fluor-488 conjugated anti-pSTAT3 (BD Bioscience). Assessment was realized by flow-cytometry.

### LSK Cell Culture in the Presence of Conditioned Media (CM)

BM-derived white blood cells were resuspended at 5 × 10^5^ cells/ml in RPMI media complemented with 10% FBS, 10 mM Hepes, 2 mM l-glutamine, 100 U/ml penicillin, 100 μg/ml streptomycin, and 0.1 mM β-mercaptoethanol. Cells were stimulated with PBS or CLCF1 for 4 h, washed, then cultured in fresh RPMI media for 24 h. The following day, CM was collected prior to the assessment of LSK frequency by flow-cytometry. CM was further incubated with freshly isolated BM cells for 24 h and LSK frequency was assessed by flow cytometry as described above.

### Cytokine and Chemokine Quantification

BM-derived cells were stimulated with recombinant murine CLCF1 (0, 10, 50, 100, 1,000 ng/ml) and incubated at 37°C for 24 h. Cell supernatants were collected and analyzed using the 31-plex mouse cytokines/chemokines arrays (Eve Technologies Corporation, Calgary, AB).

### Analysis of Hematopoiesis in Mice Undergoing CLCF1 Administration

Three sets of *in vivo* experiments were conducted. In the first setting, female 6–8 weeks old C57BL/6 mice (*n* = 10/group) received 5 daily intra-peritoneal (i.p.) injections of PBS or CLCF1 (50 or 300 μg/kg). Splenocytes, blood and BM cells were harvested from mice sacrificed at day 8 and quantified by flow-cytometry. BM cells were stained for 1 h on ice with PBS containing 0.1% BSA, V450- conjugated -anti-Lineage Cocktail, PercPCy5.5-conjugated anti-CD117 (c-Kit), and APC-Cy7 conjugated anti-Ly-6A/E (Sca1) (all purchased from BD Bioscience). Splenocytes, blood cells and BM cells were stained in PBS containing 0.1% BSA, APC-eFluor7 -conjugated anti-CD11b, FITC-conjugated anti CD19 and eFluor 450-conjugated anti-CD3 (all purchased from ThermoFisher Scientific Inc.).

In the study concerning the effect of CLCF1 on mice undergoing sub-lethal irradiation, female 6–8 weeks old C57BL/6 mice (*n* = 10 per group) were subjected to 5 Gy irradiation 24 h prior to treatments using a Faxitron RX-650 irradiator. PBS or CLCF1 (100 μg/kg) were administered i.p. daily for 5 consecutive days. Five mice per group were sacrificed at day 8 or 28 after irradiation and BM-derived LSKs were quantified by flow-cytometry as described above. BM cells were stained with PE-conjugated anti-CD11b and APC-conjugated anti-CD19 (BD Bioscience). Fluorescence was quantified using a FacsCanto II flow cytometer.

In the third and final setting, irradiated female C57BL/6 recipient mice (8 Gy; *n* = 10/group) were transplanted with T-cell-depleted 2 × 10^6^ B6.SJL-derived BM (CD45.1^+^ → CD45.2^+^). On the following day, mice were i.p.-injected with CLCF1 (300 μg/kg), IL-7 (50 μg/kg), or equivalent volume of PBS every 2 days over a total period of 2 weeks (7 injections altogether). Immune reconstitution was assessed weekly starting a week 2 using both the Scil vet ABC Plus^+^ hematological analyzer (Scil animal care company GmbH, Gurnee, Il) and flow-cytometry on collected blood samples. Mice were sacrificed at weeks 4 and 9 for analysis of their BM and spleen compartments. Splenocytes and blood cells and BM cells were stained with PBS containing 0.1% BSA, APC conjugated anti-CD45.2, PE conjugated anti- CD45.1, APC-efluor780-conjugated anti-CD11b, FITC-conjugated anti CD19, and eFluor 450-conjugated anti-CD3 (all purchased from ThermoFisher Scientific Inc.). HSC cells were quantified using PE conjugated anti- CD45.1, V450 conjugated-anti-Lineage Cocktail, PercPCy5.5-conjugated anti-CD117 (c-Kit) and APC-Cy7 conjugated anti-Ly-6A/E (Sca1), PEcyanine7-conjugated anti-CD48 (all from BD Bioscience), and APC-conjugated anti-CD150 (clone 9D1, ebiosciences, ThermoFisher Scientific Inc., Burlington, ON).

### Statistical Analysis

*P*-values were calculated using one way analysis of variance (ANOVA) with Bonferroni *post hoc* test where appropriate. Results are represented as average mean with S.D. error bars, and statistical significance is represented with asterisks: ^*^P < 0.05, ^**^P < 0.01, ^***^P < 0.001.

## Results

### CLCF1 Maintains LSK Cells *in vitro*

**Figure 1:**
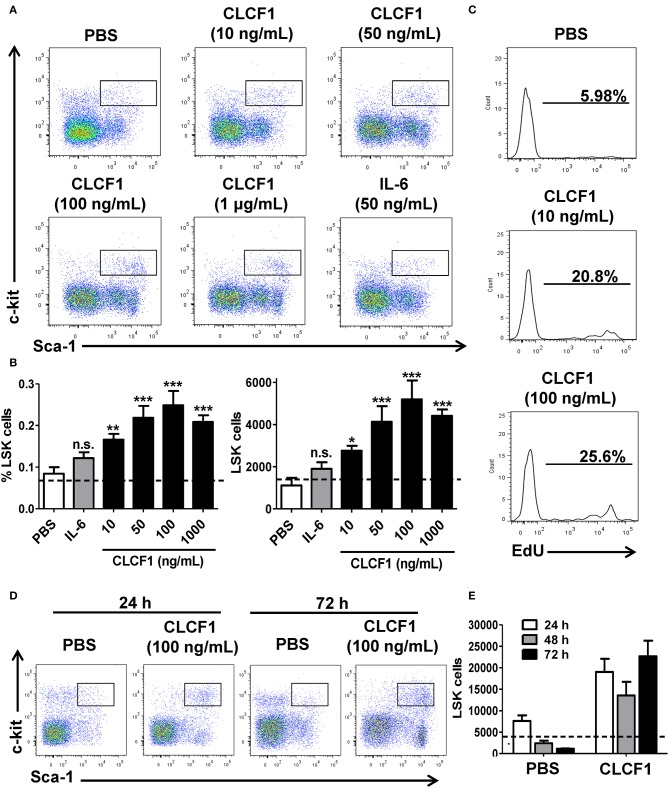
CLCF1 promotes LSK proliferation. **(A)** Representative flow-cytometry analysis of LSKs following a 24 h stimulation with different CLCF1 concentrations. **(B)** Percentages and absolute counts of LSKs displayed as mean of triplicates ± S.D. Dotted lines represent LSK counts at day 0. **(C)** EdU incorporation in LSK cells after 24 h of WBM stimulation with CLCF1. **(D)** Flow-cytometry analysis of LSKs following 24 and 72 h stimulation with PBS or CLCF1 (100 ng/mL). **(E)** Absolute LSK counts of the experiment shown in **(D)**. Dotted lines represent LSK counts at day 0. ^*^P < 0.05, ^**^P < 0.01, ^***^P < 0.001.

We first evaluated the capacity of CLCF1 in triggering HSC and early progenitor expansion *in vitro*. The whole BM (WBM) collected from C57BL/6 mice was incubated with increasing concentrations of CLCF1 for 24, 48, or 72 h. Addition of 10–1000 ng/ml of CLCF1 for 24 h increased both LSK frequency and absolute counts (Figures 1A,B), which was further confirmed by EdU incorporation (Figure 1C). The observed increase in LSK cells was maintained up to 72 h indicating a long-lasting effect of CLCF1 in limiting cell differentiation and/or maintaining self-renewal (Figures 1D,E). LSK functionality at 24 h was assessed using colony forming unit assays in methylcellulose. Results indicate that LSK cells from CLCF1-treated BM cultures formed significantly more colonies than PBS-treated cultures (Figure S1). Phenotypic analysis of LSK for SLAM marker, CD16/32, CD41, and CD105 expression suggests that CLCF1 specifically increases MPP counts (Figure S2). Altogether, these results reveal a capacity for CLCF1 in maintaining LSK cell functionality *in vitro*.

### The Effect of CLCF1 on LSK Cells *in vitro* Involves Soluble Mediators

**Figure 2:**
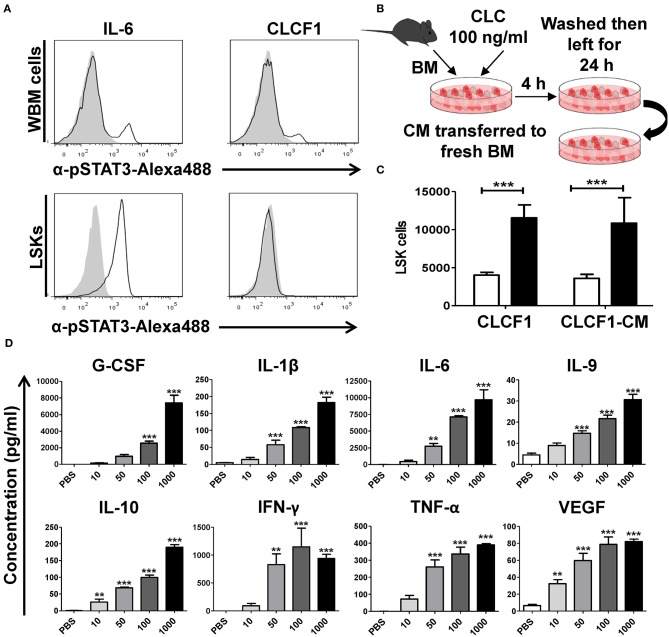
CLCF1 activates LSK cells in an indirect fashion. **(A)** WBM (upper panel) or FACS-sorted LSKs (lower panel) stimulated with IL-6 (50 ng/ml) or CLCF1 (100 ng/ml). Filled gray histograms represent STAT3 phosphorylation in unstimulated cells. **(B)** The experimental design used to assess the indirect effect of CLCF1 on BM-derived cell. WBM cells were incubated with PBS or CLCF1 for 4 h, washed then cultured for another 24 h. CM was then transferred to stimulate freshly isolated BM cells for 24 h prior to LSK quantification by flow-cytometry. **(C)** Absolute LSK counts from the experiment shown in panel B. CLCF1 or CLCF1-derived CM is represented by the black bars whereas white bars represent the PBS control condition. **(D)** Cytokine/chemokine analysis of CM collected from cells incubated with PBS or CLCF1 for 24 h. ^**^P < 0.01, ^***^P < 0.001.

The effect of CLCF1 on neurons, skeletal muscle cells and podocytes are believed to be exhibited by the recruitment of the heterotrimeric CNTF receptor comprising CNTFRα, LIFRβ, and gp130 (11). The net outcome culminates in JAK/STAT-mediated activation of STAT1 and STAT3. However, CNTFRα is absent on immune cells suggesting an alternative CLCF1 mechanism of action at play (15–18). To unveil the CLCF1 mode of action on LSK cells, we compared STAT3 phosphorylation in both WBM and sorted LSK cells in response to *in vitro* CLCF1 stimulation. Interestingly, both CLCF1 and IL-6 triggered STAT3 phosphorylation in WBM cells (Figure 2A, upper panel), whereas CLCF1, unlike IL-6, failed at inducing STAT3 activation in sorted LSK cells (Figure 2A, lower panel). These results suggest an indirect CLCF1-mediated effect on LSKs. To ascertain this hypothesis, we designed an *in vitro* experiment where CM was collected from CLCF1-primed WBM cells and then used to stimulate freshly isolated WBM (Figure 2B). This experiment revealed that CM derived from CLCF1-treated WBM could expand LSKs to the same extent as direct CLCF1 stimulation (Figure 2C). This prompt us to analyze the supernatants collected from WBM cells stimulated for 24 h with increasing concentration of CLCF1 using a broad cytokine array. Interestingly, CLCF1 triggered the up-regulation of various factors known to regulate hematopoiesis in a dose-dependent manner (Figure 2D; Figure S3). Altogether, these results demonstrate that CLCF1 mediates it effect on LSK cells *in vitro* via the upregulation of pro- and anti-inflammatory cytokines.

### CLCF1 Administration to Healthy Animals Increases the Frequency and Count of LSK and Myeloid Cells

**Figure 3:**
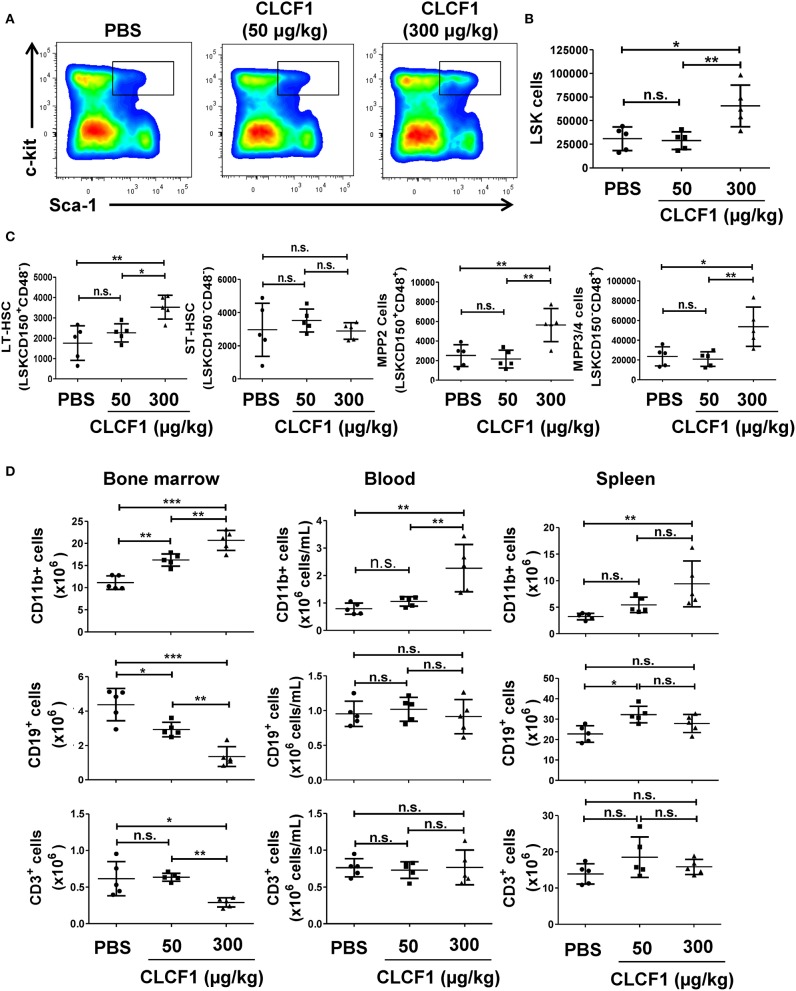
CLCF1 administration to healthy mice up-regulate LSK and myeloid cell levels in the BM. **(A,B)** Frequency and count assessment of LSKs at day 8 post-CLCF1 administration. **(C)** Absolute counts of BM LSKsCD150^+^CD48^−^ (LT-HSCs), LSKsCD150^−^CD48^−^ (ST-HSCs), LSKsCD150^+^CD48^+^ (MPP2), and LSKsCD150^−^CD48^+^ (MPPs). **(D)** Absolute counts of BM-, blood- and spleen-resident CD11b^+^ cells, CD19^+^ cells and CD3^+^ cells at day 8. Graphs represent absolute count in mean ± S.D (*n* = 5 per group). ^*^P < 0.05, ^**^P <0.01, ^***^P < 0.001.

Having observed that CLCF1 can induce LSK cell proliferation *in vitro*, we next sought to examine the effect of CLCF1 on hematopoiesis *in vivo*. To achieve this objective, we injected 50 or 300 μg/kg of recombinant CLCF1 into C57BL/6 mice daily for 5 consecutive days prior to analyzing the BM, blood and spleen at day 8 post-CLCF1. Administration of 300 μg/kg of CLCF1 resulted in a marked increase in the frequency (Figure 3A) and absolute count (Figures 3B,C) of BM-derived LSKs. Interestingly, a consistent increase in CD11b^+^ cells in the BM, blood and spleen compartments was also noticed (Figure 3D), in contrast to B (CD19^+^) and T (CD3^+^) cells, clearly indicating a preferential effect for CLCF1 on myelopoiesis.

### CLCF1 Accelerates LSK and Myeloid Cell Recovery Following Sub-lethal Irradiation

**Figure 4:**
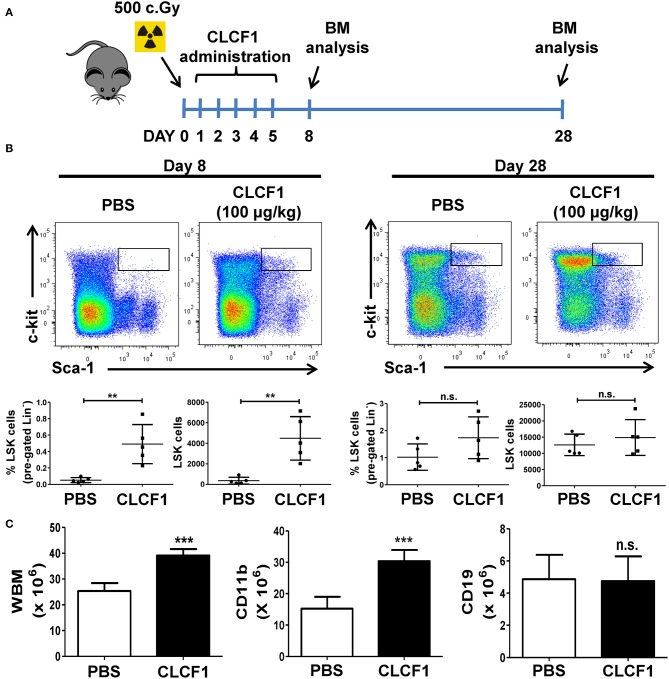
CLCF1 accelerates the recovery of LSKs following sub-lethal irradiation. **(A)** Schematic overview of CLCF1 injections after total body sub-lethal irradiation. **(B)** Flow-cytometry analysis and their cognate percentages/absolute counts of BM-derived LSK cells at day 8 (left panel) or day 28 (right panel) compared to PBS-treated mice. **(C)** Absolute count of total, CD11b^+^ and CD19^+^ BM cells at day 28. For **(B,C)**, graphs represent absolute count in mean ± S.D (*n* = 5 per group). ^**^P < 0.01, ^***^P < 0.001.

Having assessed the effect of CLCF1 on hematopoiesis in healthy animals, we then evaluated the capacity of CLCF1 in regulating LSK expansion during emergency hematopoiesis induced by sub-lethal total body irradiation. Under such condition, hematopoiesis recovery usually occurs gradually to reach normal levels in mice 1 month after the insult is cleared (19). This model is therefore suitable to assess the speed of hematopoiesis recovery following CLCF1 administration. CLCF1 was administered as previously explained prior to BM analysis at days 8 and 28 post-irradiation (Figure 4A). Interestingly, we observed more than a 10-fold increase in the absolute number of LSKs at day 8 compared to PBS treated mice (Figure 4B). Since recovery occurs gradually in this model, no significant differences in LSK counts were observed at day 28 (Figure 4C). Of note, higher counts of total BM and CD11b^+^ cells were detected at day 28 in the CLCF1-treated group (Figure 4D) further supporting a role for CLCF1 in myeloid-based hematopoiesis.

### CLCF1 Promotes Myelopoiesis Following Congenic BMT

**Figure 5:**
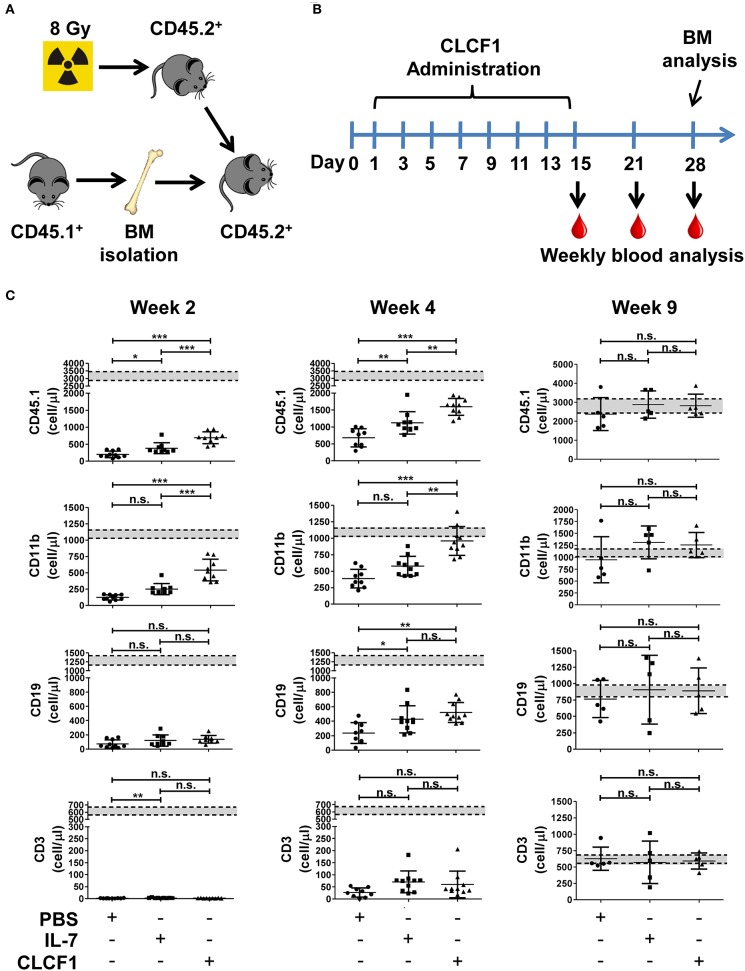
CLCF1 administration increases the level of circulating myeloid cells following congeneic BMT. **(A,B)** Schematic representation of CLCF1 injections in congenic BMT. Recipient mice received i.p. injections of PBS, IL-7 (50 μg/kg) or CLCF1 (300 μg/kg) every 2 days for a total period of 2 weeks. Blood samples were collected weekly starting from week 2 for flow-cytometry analysis. **(C)** Absolute counts of circulating CD45.1^+^, CD45.1^+^CD11b^+^, CD45.1^+^CD19^+^, and CD45.1^+^CD3^+^ cells at week 2, 4, and 9 post-transplantation. Graphs represent absolute counts in mean ± S.D (*n* = 10/group for week 2 and 4, *n* = 5/group for week 9). Dotted lines indicate mean value of aged-match un-transplanted controls (*n* = 10) ±S.D. ^*^P < 0.05, ^**^P < 0.01, ^***^P < 0.001.

**Figure 6:**
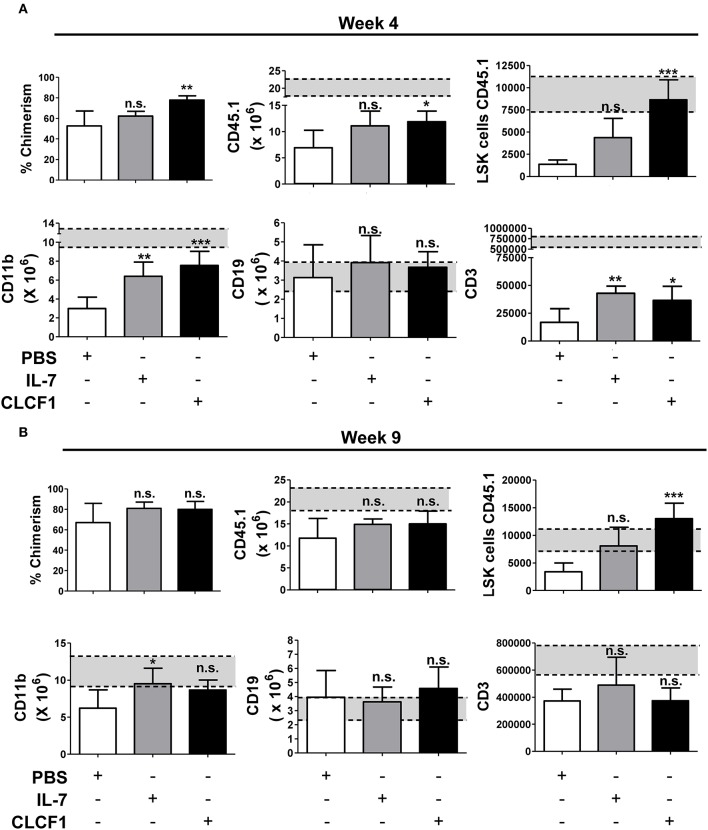
CLCF1 administration after BMT results in increased numbers of BM-resident LSK and myeloid cells. Recipient mice received i.p. injections of PBS, IL-7 (50 μg/kg), or CLCF1 (300 μg/kg) every 2 days for 2 weeks. Five mice per group were sacrificed at week 4 after congenic transplant and 5 mice at week 9 for BM flow-cytometry analysis. Percentages of chimerism (CD45.1/total CD45) and absolute counts of BM CD45.1^+^, CD45.1^+^LSKs, CD45.1^+^CD11b^+^, CD45.1^+^CD19^+^, and CD45.1^+^CD3^+^ cells at week 4 **(A)** or week 9 **(B)**. Bar graphs representing the absolute counts in mean ± S.D (*n* = 5 per group). ^*^P < 0.05, ^**^P < 0.01, ^***^P < 0.001.

Congenic BMTs are widely used models for studying HSC engraftment and immune reconstitution (20, 21). To further confirm that CLCF1 enhances LSK expansion with a bias toward myeloid cell regeneration, CLCF1 was administered to mice undergoing congeneic BMT (Figures 5A,B). An accelerated recovery of circulating CD45.1^+^ cells with a very rapid reconstitution of circulating myeloid cells was observed (Figure 5B). Significant differences were noticed between CLCF1- and PBS-treated mice as soon as 2 weeks post-transplantation (Figure 5C). In fact, CLCF1-treated mice had circulating counts of CD11b^+^ cells at week 2 equivalent to those observed at week 4 in the PBS-treated mice, with almost complete recovery reached at week 4 post-BMT (Figure 5C). Although CLCF1 was initially identified as a B-cell stimulating factor, only a mild B-cell reconstitution effect was observed with no significant impact on T-cell reconstitution at the tested doses (Figure 5C). In addition, BM analysis at week 4 showed a higher percentage of chimerism in CLCF1-treated mice compared to the PBS suggesting enhanced engraftment of progenitor cells in presence of CLCF1 (Figure 6A). In agreement with the data obtained on circulating CD45.1^+^ cells, the BM of CLCF1-treated mice displayed increased absolute counts of total CD45.1^+^ and CD11b^+^ cells (Figure 6A). Notably, higher levels of donor LSKs were detected in the CLCF1-treated mice when compared to control untreated mice at week 4 (Figure 6A; Figure S4). Interestingly, the increase of donor LSKs in response to CLCF1 was maintained at week 9 (Figure 6B; Figure S4). Altogether, our observations using the congenic BMT model confirm the hematopoiesis-stimulating properties of CLFC1 with a myelopoiesis bias.

## Discussion

We observed a new role for CLCF1 in stimulating LSK proliferation with a bias toward myeloid cell development. Amongst our observations, the high LSK counts observed after a 72 h culture with CLCF1 begs the question: how can CLCF1 restrain LSK cell differentiation? In fact, analysis of BM following congenic BMT revealed increased counts in long term (LT)-HSCs and multipotent progenitor cells suggesting both sustained hematopoiesis and a rapid differential potential, respectively (Figure S4). A logical explanation for these observations may lay in the CLCF1 mode of action. Analysis of CM collected in response CLCF1-primed WBM cells revealed the presence of well-described soluble factors known for their role in hematopoiesis. Among the detected factors, the presence of IL-10 might be of capital importance due to its involvement in promoting HSC self-renewal (22). Furthermore, sustained hematopoiesis has been also linked to VEGF activity whereas its genetic ablation results in reduced HSC survival and colony formation capacity (23). *Ex-vivo* stem cell expansion is nowadays of high clinical interest but its success depends largely on the quality and amount of injected HSCs (24). Although beyond the scope of this study, it would be pertinent to assess whether CLCF1 could improve *ex-vivo* HSC expansion or accelerate HSC engraftment when combined with established cytokines and growth factors enrichment cocktails or small molecules.

Myelopoiesis is an important and tightly regulated process insuring sustained formation of monocytes, granulocytes and dendritic cells. Myeloid cells are important players in innate and adaptive immunity and macrophages are primordial for the resolution of inflammation. Our data bring forward new assumptions and hypotheses, which could be of interest for immunotherapies and/or pathologies involving myeloid dysregulation. For instance, BCG vaccination has been recently shown to educate HSCs in order to generate epigenetically-modified macrophages capable of providing better protection against virulent *M. tuberculosis* infection compared to standard naïve macrophages (25). It would therefore be interesting to: (i) assess CLCF1 expression following BCG administration, and (ii) test whether its co-administration with BCG generates an optimal protective innate immunity against tuberculosis. On a different subject, HSC reconstitution during aging is altered by clonal expansion leading to a myeloid dominance during hematopoiesis (26–28). Cytokines secretion following CLCF1 treatment comprises factors associated with the inflammatory profile of aging. In fact, both IL-6 and TNF-α have been reported to be elevated in elderly populations and are strongly secreted in BM cultures stimulated with CLCF1 (Figure 2D) (26). This suggests that CLCF1 might contribute in promoting myeloid cell development during aging and could therefore represent a potential therapeutic target for the re-establishment of a balanced myelo-/lymphopoiesis. Further studies on hematopoiesis and CLCF1 are therefore warranted to evaluate the clinical relevance of this cytokine.

## Ethics Statement

This study was carried out in accordance with the recommendations of the Canadian Council on Animal Care guidelines, Animal Ethics Committee of Université de Montréal. The protocol was approved by the Animal Ethics Committee of Université de Montréal.

## Author Contributions

SP designed and performed *in vitro* experiments, proliferations, STAT3 phosphorylation assays, conditioned media experiments, realized CLCF1 administration in the three set of *in vivo* experiments, analyzed data, and wrote the paper. AT designed and helped with the sub-lethal total body irradiation assays. JM and VL participated in CLCF1 production and congeneic bone marrow transplant experiments. MS participated in study concept, analysis, and interpretation of data. J-FG and MR conceived and supervised the project, analyzed data, and wrote the paper. All authors contributed to the conception of the study, the analysis of the data, and the revision of the manuscript.

### Conflict of Interest Statement

AT was employed by company Immuni T. The remaining authors declare that the research was conducted in the absence of any commercial or financial relationships that could be construed as a potential conflict of interest.
