# Supplementary material for: Cardiotrophin-Like Cytokine Factor 1 Exhibits a Myeloid-Biased Hematopoietic-Stimulating Function
Source: Front Immunol. 2019 Sep 10;10:2133. doi: 10.3389/fimmu.2019.02133 (PMC6746841; doi:10.3389/fimmu.2019.02133)
Supplement: Supplementary file 1 [file Presentation_1.pdf]

Supplementary Material

Cardiotrophin-Like Cytokine Factor 1 Exhibits a Myeloid-Biased Hematopoietic-Stimulating Function

Sarah Pasquin,[1] Aurélie Tormo,[1,2] Jessica Moreau,[1] Véronique Laplante,[1] Mukut Sharma,[3] Jean-François Gauchat[1*] and Moutih Rafei[1,4,5,6*]

[*]To whom correspondence should be addressed:
Dr. Moutih Rafei:
moutih.rafei.1@umontreal.ca;

Dr. Jean-François Gauchat:
jf.gauchat@umontreal.ca.

Supplementary Figures

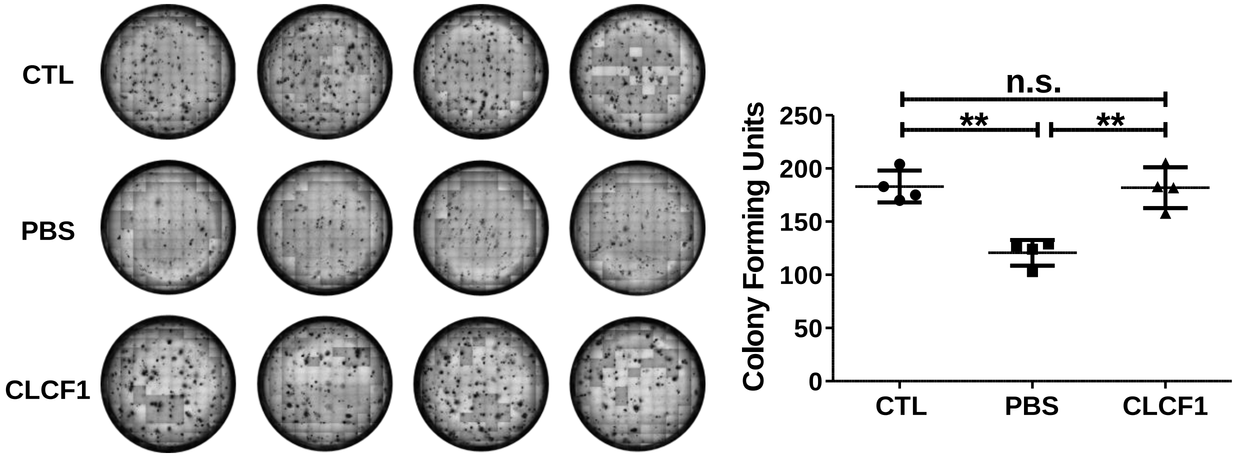

Figure S1. CLCF1 sustains the *in vitro* formation of clonogenic progenitors. Representative images (left) and quantification analysis (right) of colony-forming units (CFU) obtained using freshly isolated BM cells (CTL), BM cells incubated with PBS or CLCF1 (100 ng/ml) for 24 h. BM cells were plated in equal proportions per condition and cultured for 7 days in methylcellulose-based complete medium (Methocult GF M3434). Bars represent mean of triplicates ± S.D. ** $P < 0.01$.

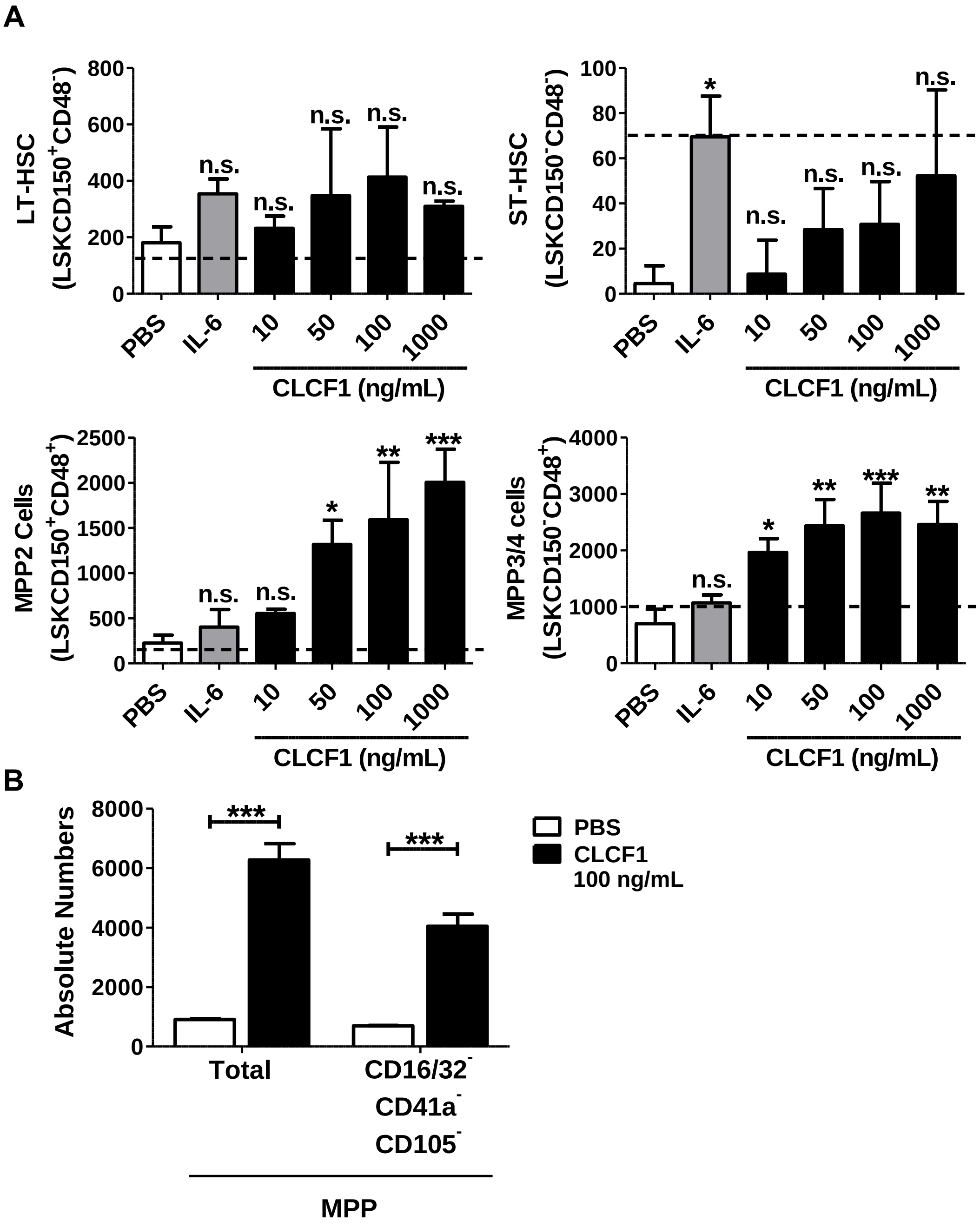

Figure S2. CLCF1 promotes MPP cells expansion *in vitro*. **(A)** Absolute counts of LT-HSC are defined as LSKCD150$^+$CD48$^-$ cells, ST-HSC as LSKCD150$^-$CD48$^-$ cells, MPP2 as LSKCD150$^+$CD48$^+$ cells and MPP3/4 as LSKCD150$^-$CD48$^+$ following a 24 h stimulation with different CLCF1 concentrations. **(B)** MPP cells (LSKCD48$^+$) from BM cultures treated with PBS or CLCF1 (100 ng/ml) were further analysed using anti-CD16/32, anti-CD41 and anti-CD105 to exclude potential progenitor cells populations. Bars represent mean of triplicates ± S.D. * $P < 0.05$, ** $P < 0.01$, *** $P < 0.001$.

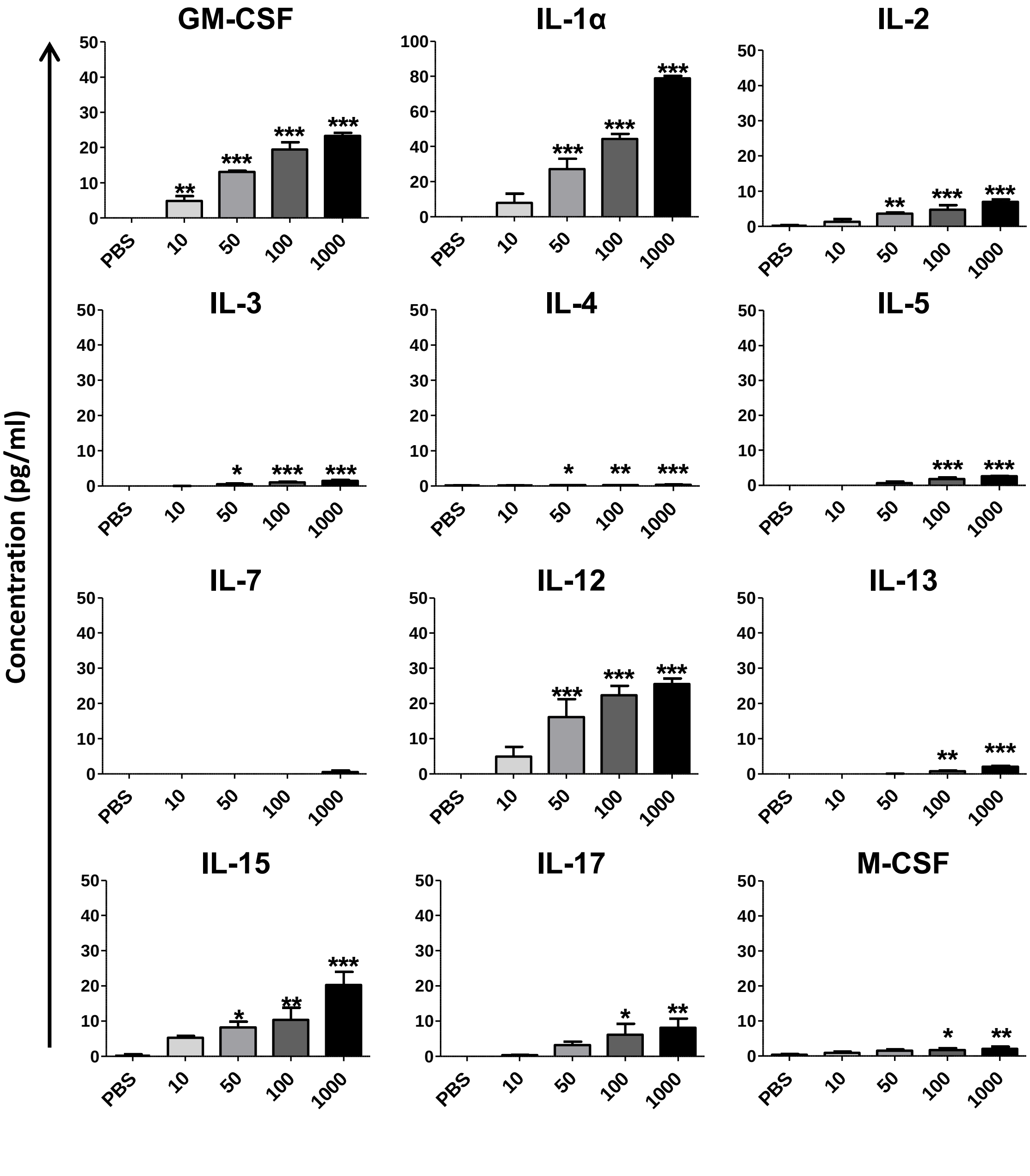

Figure S3. Quantification of cytokines secreted by BM cells stimulated with CLCF1. Cell supernatants of BM cells stimulated with PBS or CM collected from WBM cells stimulated by CLCF1 were analyzed by cytokines/chemokines arrays. Bars represent mean concentration of indicated cytokine or chemokine in pg/mL ± S.D. * $P < 0.05$, ** $P < 0.01$, *** $P < 0.001$.

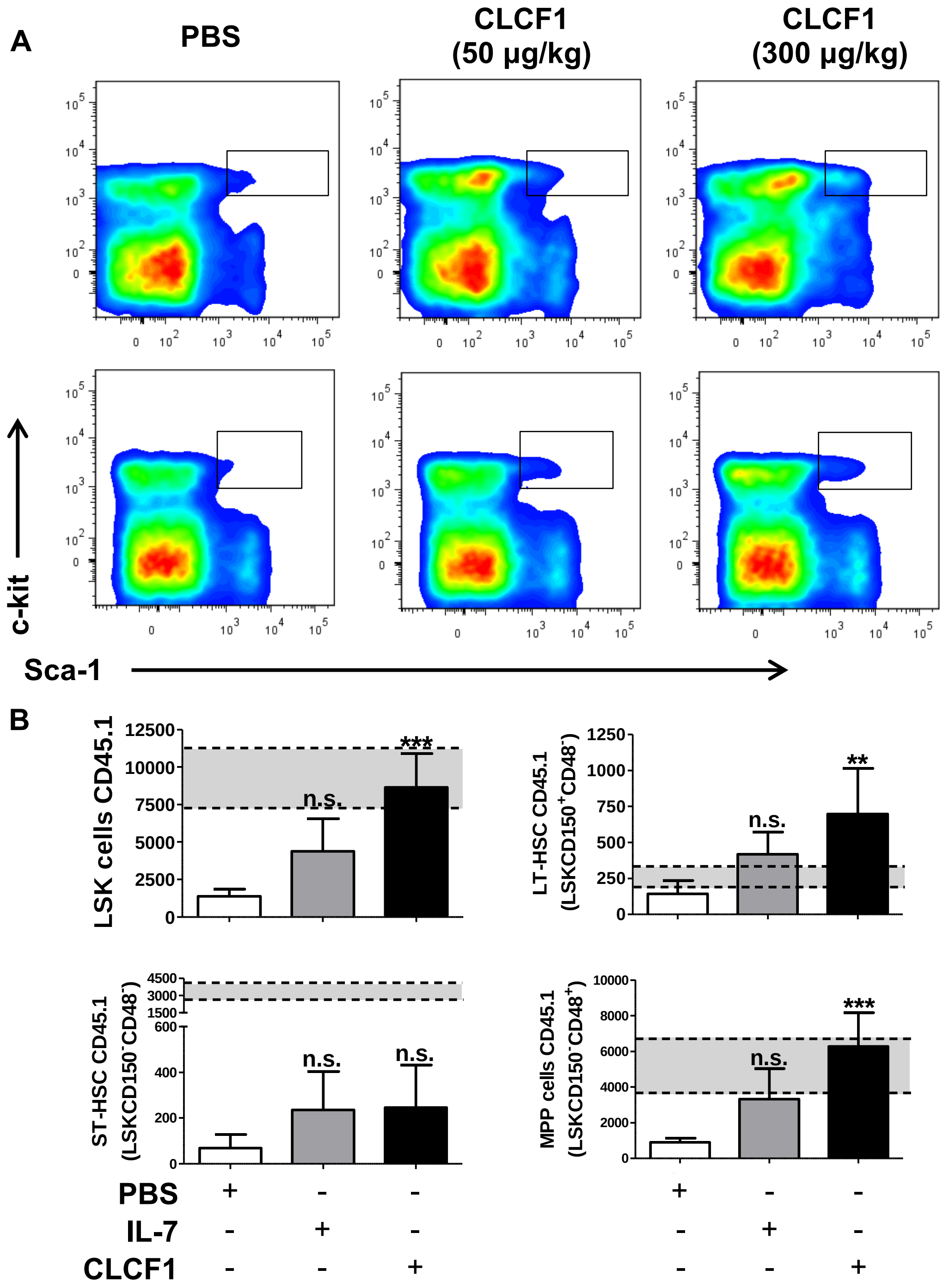

Figure S4. CLCF1 administration following congenic BMT results in increased numbers of LT-HSC and MPP cells. Recipient mice received i.p. injections of PBS, IL-7 (50 µg/kg) or CLCF1 (300 µg/kg) every second day for two weeks. Five mice per group were sacrificed at week 4 after congenic transplantation for BM analysis by flow-cytometry. (A) Representative flow cytometry analysis of LSK cells at week 4 (upper panel) and week 9 (lower panel). (B) Absolute counts of BM CD45.1$^+$LSKs, CD45.1$^+$LSKsCD150$^+$CD48$^-$ (LT-HSCs), CD45.1$^+$LSKsCD150$^-$CD48$^-$ (ST-HSCs) and CD45.1$^+$LSKsCD150$^-$CD48$^+$ (MPPs) were assessed at week 4. Bar graphs represent absolute count in mean ± S.D (n=5). ** $P < 0.01$, *** $P < 0.001$.
